# Retraction Notice to: Inhibition of tumor invasion and metastasis by targeting TGF-β-Smad-MMP2 pathway with Asiatic acid and Naringenin

**DOI:** 10.1016/j.omton.2026.201195

**Published:** 2026-04-04

**Authors:** Guang-Yu Lian, Qing-Ming Wang, Thomas Shiu-Kwong Mak, Xiao-Ru Huang, Xue-Qing Yu, Hui-Yao Lan

## Main text

(Molecular Therapy: Oncolytics *20*, 277–289; March 2021)

*Molecular Therapy Oncology* is retracting this article at the request of the editor-in-chief. A third party communicated concerns of image reuse to the editorial office. Concerns were also posted to a Pubpeer thread: https://pubpeer.com/publications/7FF4B8E8268CD0272F25323C155753. Image analysis performed by the editorial office revealed evidence of image duplication in identical or altered fashion within Figures 2A and 2B of this article. This reuse (and in part misrepresentation) of data without appropriate attribution represents a severe abuse of the scientific publishing system.

